# Grouper TRADD Mediates Innate Antiviral Immune Responses and Apoptosis Induced by Singapore Grouper Iridovirus (SGIV) Infection

**DOI:** 10.3389/fcimb.2019.00329

**Published:** 2019-09-18

**Authors:** Xin Zhang, Zetian Liu, Chen Li, Ya Zhang, Liqun Wang, Jingguang Wei, Qiwei Qin

**Affiliations:** ^1^Joint Laboratory of Guangdong Province and Hong Kong Region on Marine Bioresource Conservation and Exploitation, College of Marine Sciences, South China Agricultural University, Guangzhou, China; ^2^Guangdong Provincial Key Laboratory for Aquatic Economic Animals, Sun Yat-sen University, Guangzhou, China; ^3^Laboratory for Marine Biology and Biotechnology, Qingdao National Laboratory for Marine Science and Technology, Qingdao, China

**Keywords:** grouper, TRADD, SGIV, virus replication, apoptosis

## Abstract

Tumor necrosis factor (TNF) receptor type 1-associated DEATH domain protein (TRADD) is a TNFR1-associated signal transducer and an essential component of the TNFR1 complex that is involved in activating both apoptotic and nuclear factor (NF)-κB pathways as an adaptor. It also is required for TNFR-1-initiated neuronal apoptosis following *in vitro* infection with virus as an essential component of the antiviral response. To date, few studies have investigated the function of TRADD in lower vertebrates and its antiviral response to DNA virus infection. In the present study, a TRADD gene (named as *EcTRADD*) from the orange-spotted grouper (*Epinephelus coioides*) was cloned and characterized. The full-length cDNA of EcTRADD consists of 1,370 base pairs (bp) and contains a 44 bp 5′-terminal untranslated region (UTR), a 450 bp 3′-UTR including a poly (A) tail, and an 876 bp open reading frame encoding a putative 291 amino acid protein. EcTRADD has two conserved domains of N-terminal domain (TRADD-N) and a death domain (DD). EcTRADD was detected in all examined tissues. EcTRADD was up-regulated in the spleen after infection with Singapore grouper iridovirus (SGIV). Subcellular localization analysis revealed that EcTRADD and EcTRADD-DD exhibited a clear pattern of discrete and interconnecting cytoplasmic filaments resembling the death-effector filaments, while EcTRADD-N was observed in the cytoplasm. After infection with SGIV, EcTRADD, and EcTRADD-DD were transferred to the nucleus. Overexpression of EcTRADD and its domains inhibited replication of SGIV *in vitro*. Both EcTRADD and EcTRADD-DD induced the caspase-dependent apoptosis in control and infected cells, while EcTRADD-N inhibited the apoptosis. Additionally, EcTRADD and EcTRADD-DD significantly promoted activation of NF-κB and reporter gene p53, whereas EcTRADD-N had no significant effect on p53. The results may provide new insights into the role of fish TRADD in fish virus infection.

## Introduction

Apoptosis, or programmed death, is a complicated process of cell-regulated destruction (Hengartner, 2000). Tumor necrosis factor (TNF) can induce apoptosis and activate nuclear factor (NF)-κB, which activates both cell death and cell survival mechanisms simultaneously through signaling cascades emanating from TNF receptor 1 (TNFR1) (Rath and Aggarwal, 1999). TNFR1 contains a conserved death domain (DD) that binds to so-called death receptors and triggers the caspase cascade reaction to induce apoptosis (Olivier and Jürg, 2003).

Tumor necrosis factor receptor type 1-associated DEATH domain protein (TRADD) is a TNFR1-associated signal transducer and an essential component of the TNFR1 complex that is involved in activating both pathways by recruiting other members of the complex to the receptor (Hsu et al., 1995, 1996b). Among these members are receptor-interacting protein and Fas-associated DD protein that bind the COOH-terminal DD of TRADD and form the so-called death-inducing signaling complex (DISC) that delivers a powerful and rapid pro-apoptotic signal (Hsu et al., 1996a,b; Michael et al., 2002; Olivier and Jürg, 2003). The receptor complexes can mediate recruitment and activation of procaspase-8, which activates downstream caspases such as caspase-3 that participate in execution of the apoptotic process (Olivier and Jürg, 2003). Caspase-8 can be recruited into complexes with members of the TNF-receptor superfamily and form death effector filaments (DEF) through the death effector domain (DED) structure to trigger apoptosis (Siegel et al., 1998). TRADD occupation not only induces these pathways but also binds the NH_2_-terminal domain portion of TNF receptor associated factor 2, leading to triggering of NF-κB signaling pathways (Hsu et al., 1996b; Olivier and Jürg, 2003). However, activation of NF-κB mediates expression of the inhibitor of caspase-8 and FLIPL, thereby inhibiting the TNF-induced apoptotic pathway (Micheau et al., 2001).

Apoptotic cell death occurs widely in viral infection (Penny and Tyler, 2009; Kinpara et al., 2013). Activation of the c-Jun N-terminal kinase, NF-κB, and p53 pathways can regulate viral induced apoptosis (Kinpara et al., 2013; Guo et al., 2016a). Singapore grouper iridovirus (SGIV), the main pathogen of grouper and seabass, can cause enlargement with hemorrhage of spleen and multifocal areas of splenic degeneration of infected diseased fish (Qin et al., 2002, 2003; Huang et al., 2004). Our previous study showed that SGIV induced typical apoptosis in fathead minnow (FHM) epithelial cells (Huang X. et al., 2011). Further analyses of host and/or SGIV genes based on FHM cells will help us to understand the pathogenesis of iridescent virus (Yan et al., 2013; Wei et al., 2015; Guo et al., 2016b). Therefore, FHM cells are used in studies of SGIV infection in this paper.

Viruses, bacteria, and parasites can induce macrophage production of TNF, which binds to cell surface receptors TNFR1 or TNFR2. Trimeric TNF binds to the extracellular domain of TNFR1, triggering an intracellular signaling pathway that releases the inhibitory protein silencer of DD from the intracellular DD of TNFR1 (Hidetoshi et al., 2003). TNFR1 undergoes a conformational change to form a trimer, after which the DD recruits and binds to TRADD to regulate the NF-κB pathway for cell survival and the apoptotic pathway for cell death. Thus, there is a delicate balance between pro-apoptotic and anti-apoptotic signaling, which depends on TRADD binding to membrane-bound TNFR1 in the DISC. The outcome of receptor activation (cell survival or death) depends on the background of its activation. In some cases, inhibition of caspase cannot be blocked and may even increase TNF-induced cell death (Jones et al., 2000; Lüschen et al., 2000; Michael et al., 2002).

In some cells, p53 is required for TNFR1 dependent apoptosis (Cai et al., 1997; Ameyar et al., 1999; Rokhlin et al., 2000). Activated p53 can induce apoptotic cell death through different pathways, including the caspase-independent pathway (Chen and Wong, 2009). TRADD can shuttle between the cytoplasm and the nucleus (Michael et al., 2002) to link the TNF receptor with p53 and induce apoptosis by different mechanisms (Guo et al., 2000; Wang et al., 2001; D'Orazi et al., 2002; Michael et al., 2002; Fogal et al., 2014).

In this study, a TRADD gene from the orange-spotted grouper *Epinephelus coioides* (*EcTRADD*) was cloned and characterized. The antiviral roles of *EcTRADD* and its domains during replication of SGIV were investigated. The results will provide new insights into the function of fish TRADD against infection by fish viruses.

## Materials and Methods

### Cloning of *EcTRADD* and Bioinformatic Analysis

First-strand cDNA of grouper TRADD was synthesized from the total RNA of spleen using the SMARTTM RACE cDNA amplification kit (Clontech) for 3′ RACE and 5′ RACE. Two primers (F1 and R1) were designed (Table 1) based on the expressed sequence tag sequences of TRADD from the grouper spleen transcriptome (Huang Y. et al., 2011). PCR was performed with 10 μM of F1 or R1 and 500 nM of Nested Universal Primer A (Clontech). Denaturation was performed for 5 min at 94°C, followed by 35 cycles for 30 s at 94°C, 30 s at 60°C, and 45 s at 72°C. The PCR products of 3′ RACE and 5′ RACE were analyzed on 1% agarose gels, and extracted using an AxyPrep DNA gel extraction kit (AxyGEN), then sequenced. The sequence of the open reading frame (ORF) of EcTRADD was obtained and confirmed by replication with PCR using specific primers F2 and R2 (Table 1).

**Table 1:** Primers used in this study.

**Name**	**Sequence(5^′^-3^′^)**
C1-EcTRADD-N-F	TCAAGCTTCGATGGCAGACAAGAATGTGGATCATGGAC
C1-EcTRADD-N-R	GTGGATCCAAACTTGAAGCAGTTGCTGGGG
C1-EcTRADD-DD-F	TCAAGCTTCGATGCAGAATAAAGTGTTTGAGGACC
C1-EcTRADD-DD-R	GTGGATCCTGGCTGTAAATCCAGAATATTTTC
HA-EcTRADD-N-F	GCGGATCCATGGCAGACAAGAATGTGGATCATG
HA-EcTRADD-N-R	CGCTCGAGTCAAAACTTGAAGCAGTTGCTGGGG
HA-EcTRADD-DD-F	GCGGATCCATGCAGAATAAAGTGTTTGAGGACC
HA-EcTRADD-DD-R	CGCTCGAGTCATGGCTGTAAATCCAGAATATTT
Actin-RT-F	TACGAGCTGCCTGACGGACA
Actin-RT-R	GGCTGTGATCTCCTTCTGCA
ORF049-F	CCCGCAATGAACTCGCCAAAACT
ORF049-R	CCGTGACGTACTGCCAAGCCTGA
ORF072-F	GCACGCTTCTCTCACCTTCA
ORF072-R	AACGGCAACGGGAGCACTA
ORF086-F	ATCGGATCTACGTGGTTGG
ORF086-R	CCGTCGTCGGTGTCTATTC

The sequence of EcTRADD was analyzed using the BLAST program (http://www.ncbi.nlm.nih.gov/blast), and the conserved domains or motifs were predicted using the Conserved Domains program (https://www.ncbi.nlm.nih.gov/cdd/). Amino acid alignments were carried out using Clustal X1.83 software and edited using the GeneDoc program. The phylogenetic analysis was conducted using the neighbor-joining (NJ) method in MEGA 6.0 software.

### Fish, Cells, and Virus

Juvenile orange-spotted groupers (weight 30–40 g) used in this study were purchased from Wenchang Marine Fish Farm, Hainan Province, China. They were kept in a laboratory recirculating seawater system at 24–28°C and fed twice daily for 2 weeks.

Grouper spleen (GS) (Huang et al., 2009) and FHM epithelial (Gravell and Malsberger, 1965) cell lines were grown at 28°C in Leibovitz's L15 and M199 culture medium with 10% fetal bovine serum (Gibco, USA), respectively. Propagation of SGIV was performed as described previously (Hegde et al., 2002; Qin et al., 2003). The viral titer of SGIV was 10^5^ TCID_50_/ml.

### RNA Extraction and cDNA Synthesis

Total RNA was extracted using SV Total RNA Isolation Kit (Promega, USA) according to the manufacturer's protocol. The quality of total RNA was assessed by electrophoresis on 1% agarose gel. Total RNA was reverse transcribed to synthesize the first-strand cDNA using the ReverTra Ace kit (TOYOBO, Japan) according to the manufacturer's instructions.

### Expression Patterns for *EcTRADD* in Grouper

To elucidate the tissue distribution of *EcTRADD* in healthy orange-spotted grouper, different tissue samples including kidney, heart, liver, spleen, intestine, stomach, muscle, brain, gill, head kidney, fin, and skin from six fish were collected for RNA extraction and further real-time quantitative PCR analysis (qRT-PCR). To detect the expression profiles of EcTRADD in response to virus infection, the orange-spotted groupers were infected with SGIV and harvested at 0, 9, 12, 24, 48, and 96 h. Total RNA was extracted and the expression of *EcTRADD* was analyzed using qRT-PCR.

### Plasmid Construction and Cell Transfection

In order to illustrate the potential function and subcellular localization of *EcTRADD*, the sequences of *EcTRADD, EcTRADD-DD*, and *EcTRADD-N* were cloned into pEGFP-C1 and pcDNA3.1-3HA vectors using primers listed in Table 1, and the plasmids of *EcTRADD, EcTRADD-DD*, and *EcTRADD-N* were constructed successfully.

Cell transfection was carried out using transfection reagent (TA) Lipofectamine 2000 reagent (Invitrogen) as described previously (Xiaohong et al., 2013). Briefly, FHM and GS cells were seeded in 24-well cell culture plates or 6-well plates at 60–70% confluence, then incubated with the mixture of Lipofectamine 2000 and plasmids for 6 h and replaced with the fresh normal medium.

### Confocal Laser Scanning Microscope and Fluorescence Microscopy

The plasmids of pEGFP-C1, pEGFP-EcTRADD, pEGFP-EcTRADD-DD, and pEGFP-EcTRADD-N were transfected into FHM and GS cells as described above. Cells were fixed with 4% paraformaldehyde, and stained with 4,6-diamidino-2-phenylindole (DAPI) at 24 h post-transfection. Fluorescence was observed under confocal laser scanning microscope and fluorescence microscope (Leica, Germany).

### Virus Infection Assay

To evaluate the effects of EcTRADD on virus infection, GS cells overexpressing pEGFP-C1, pEGTP-EcTRADD, pEGFP-EcTRADD-N, or pEGFP-EcTRADD-DD were infected with SGIV at a multiplicity of infection (MOI) of 2. At indicated time points, the cell morphology was observed under a phase contrast microscopy. Meanwhile, parallel cell samples were harvested for RNA extraction and cDNA synthesis, and the expression profiles of three SGIV genes (ORF049, ORF072, and ORF086) were evaluated by qRT-PCR using primers listed in Table 1.

### qRT-PCR

qRT-PCR was performed in an Applied Biosystems Quant Studio 3 Real Time Detection System (Thermofisher, USA) to check the transcriptional expression level of host and virus genes. Each assay was carried out in triplicate with the following cycling conditions: 1 min for activation at 95°C, followed by 40 cycles for 15 s at 95°C, 15 s at 60°C, and 45 s at 72°C. The primers used were listed in Table 1. The expression levels of target genes were normalized to β-actin and calculated with the 2^−ΔΔ*CT*^ method. The data were represented as mean ± SD.

### Analysis of Cell Apoptosis

FHM cells overexpressing pcDNA3.1-3HA, pcDNA3.1-EcTRADD, pcDNA3.1-EcTRADD-N, or pcDNA3.1-DD were infected with SGIV at MOI of 2. Cells were stained with Hoechst 33342 at 12 h post-infection, and the morphologies of apoptotic corpuscles were observed under fluorescence microscope. Meanwhile, other cells were harvested, and washed twice with PBS, and resuspended in 1 × Binding Buffer at a concentration of 1 × 10^6^ cells/ml. Then 100 μl of the solution (1 × 10^5^ cells) were transferred to a 5 ml culture tube, and 5 μl of FITC Annexin V and 5 μl PI were added. The cells were gently vortexed and incubated for 15 min at 25°C in the dark. Four hundred microliter of 1 × Binding Buffer was added to each tube. Then the cells were analyzed by flow cytometry within 1 h. Data analysis was performed with FlowJo V10.

### Assays of Caspase Activation

Caspase-8 can induce classic apoptosis, which involves the activation of caspases that are associated with the assembly of cytoplasmic DEFs (Siegel et al., 1998). The activities of caspase-8 and caspase-3 were measured using IETD-AFC and DEVD-AFC (BioVision). Cells were collected from monolayers and then lysed in 60 μl of cell lysis buffer for 10 min on ice. After centrifugation, 45 μl of supernatant were extracted and added to a 96-well plate. Next, 50 μl of 2 × reaction buffer (containing 10 Mm dithiothreitol) were added to each sample. Finally, 5 μl of the 1 Mm IETE-AFC or DEVD-AFC substrates (50 μM final concentration) were added, and the mixture was incubated for 1–2 h at 37°C. Levels of cleaved caspase substrate were measured using a spectrofluorometer (Molecular Devices).

### Reporter Gene Assays

To examine the effects of EcTRADD on the activity of p53 and NF-κB promoter, luciferase plasmids including NF-κB-Luc and p53-Luc were used in this study. In brief, FHM cells were co-transfected with 200 ng NF-κB-Luc (p53-Luc) and 600 ng EcTRADD, EcTRADD-N, or Ec TRADD-DD, respectively. A total of 50 ng SV40 was included to normalize the luciferase activities. Then cells were harvested to measure the luciferase activities by Dual-Luciferase® Reporter Assay System (Promega) at 24 h post-infection according to the manufacturer's instructions.

### Statistical Analysis

Statistics were carried out using SPSS version 20 by one-way analysis of variance. Differences were considered statistically significant at ^*^P < 0.05 and ^**^P < 0.01.

### Ethics Statement

All animal-involving experiments of this study were approved by the Animal Care and Use Committee of College of Marine Sciences, South China Agricultural University, and all efforts were made to minimize suffering.

## Results

### Sequence Characterization of EcTRADD

**Figure 1:**
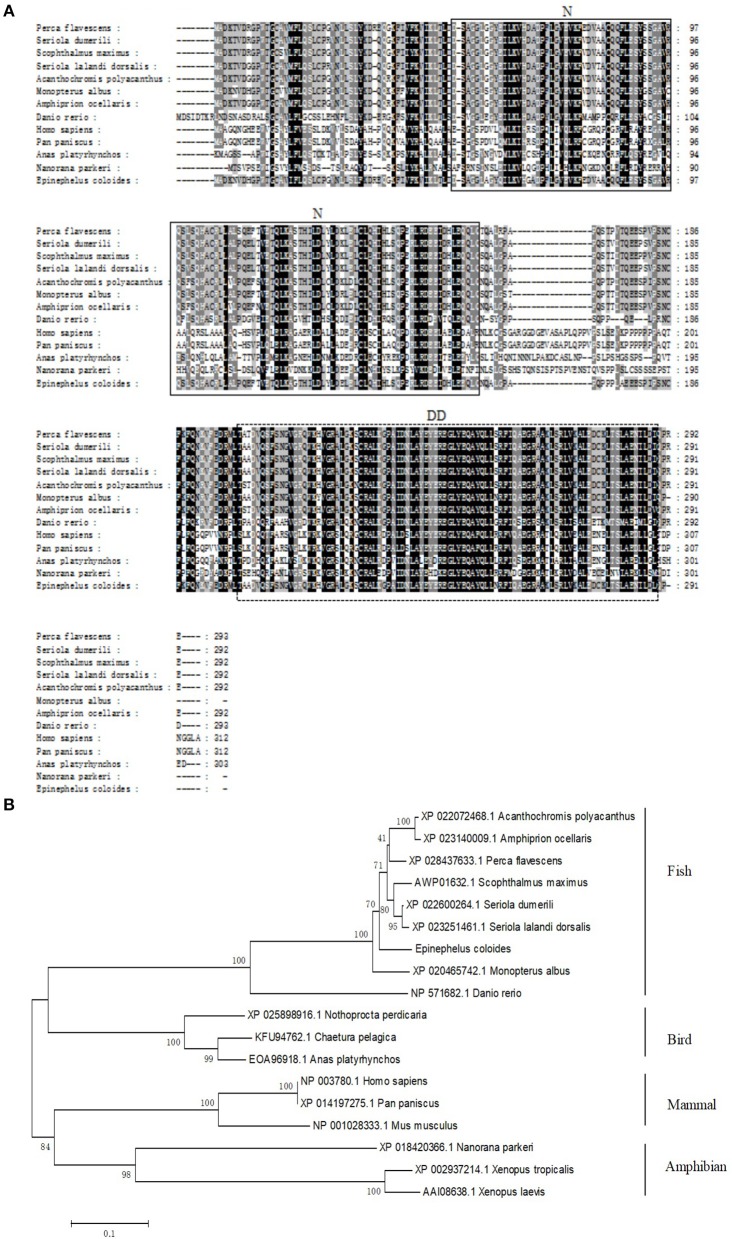
Molecular cloning of grouper TRADD. **(A)** Multiple sequence alignments of TRADDs. The centrally localized TRADD-N and DD are boxed with solid lines and dotted lines, respectively. The accession numbers are as follows: *Perca flavescens*, XP_028437633.1; *Seriola dumerili*, XP_022600264.1; *Scophthalmus maximus*, AWP01632.1; *Seriola lalandi dorsalis*, XP_023251461.1; *Acanthochromis polyacanthus*, XP_022072468.1; *Monopterus albus*, XP_020465742.1; *Amphiprion ocellaris*, XP_023140009.1; *Danio rerio*, NP_571682.1; *Homo sapiens*, NP_003780.1; *Pan paniscus*, XP_014197275.1; *Anas platyrhynchos*, EOA96918.1; and *Nanorana parkeri*, XP_018420366.1. **(B)** The phylogenetic tree was constructed according to the alignment of amino acid sequences using the neighbor-jointing method within MEGA 6.0, with 1,000 bootstrap replications. The scale represents the numbers of substitutions per 1,000 bases. The bootstrap values are indicated at the nodes of the tree. The GenBank accession number of each species is listed to the left of the species name.

Based on the partial cDNA sequences of TRADD, the full-length cDNA of *EcTRADD* was amplified. It contains a 44 bp 5′-terminal untranslated region (UTR), a 450 bp 3′-UTR including a poly (A) tail, and an 876 bp open reading frame encoding a putative 291 amino acid protein. Conserved Domains-Search (CD-Search) analysis showed that two conserved domains—TRADD-N at positions 52–162 and DD at positions 200–289—are present in *EcTRADD*. BLAST analysis revealed that EcTRADD shares 91.81% identity with the yellow perch *Perca flavescens* (XP_028437633.1). Multiple sequence alignments were carried out using Clustal X multiple-alignment software. Phylogenetic trees were made using the NJ method with 1,000 bootstraps. EcTRADD is clustered in the Osteichthyes branch and the TRADD subfamily is conservative among Osteichthyes (Figures 1A,B).

### Tissue Distribution and Expression Profiles of EcTRADD *in vivo*

**Figure 2:**
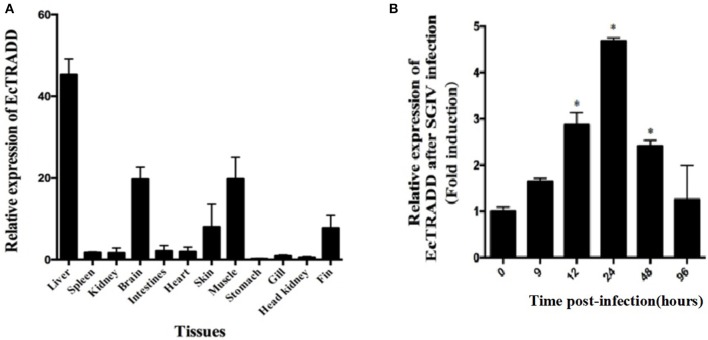
Expression patterns of EcTRADD. **(A)** Tissue distribution of EcTRADD in healthy groupers. **(B)** Expression of EcTRADD in the spleen of groupers at different time points post-SGIV infection. Error bars represent standard deviations of triplicates, and * indicates that the means were statistically significantly at *P* < 0.05.

The transcript levels of *EcTRADD* in different tissues from healthy juvenile orange-spotted groupers were analyzed using qRT-PCR. As shown in Figure 2A, *EcTRADD* was distributed in all 12 examined tissues and predominantly detected in liver, brain, muscle, skin, and fin.

To characterize the expression patterns of the *EcTRADD* gene during viral infection, the expression profiles of *EcTRADD* in spleen after SGIV infection was determined by qRT-PCR. *EcTRADD* was up-regulated in the spleen after SGIV infection; it reached the maximal level at 24 h p.i., which was 5-fold higher than that of control (Figure 2B).

### Cellular Localization of EcTRADD

**Figure 3:**
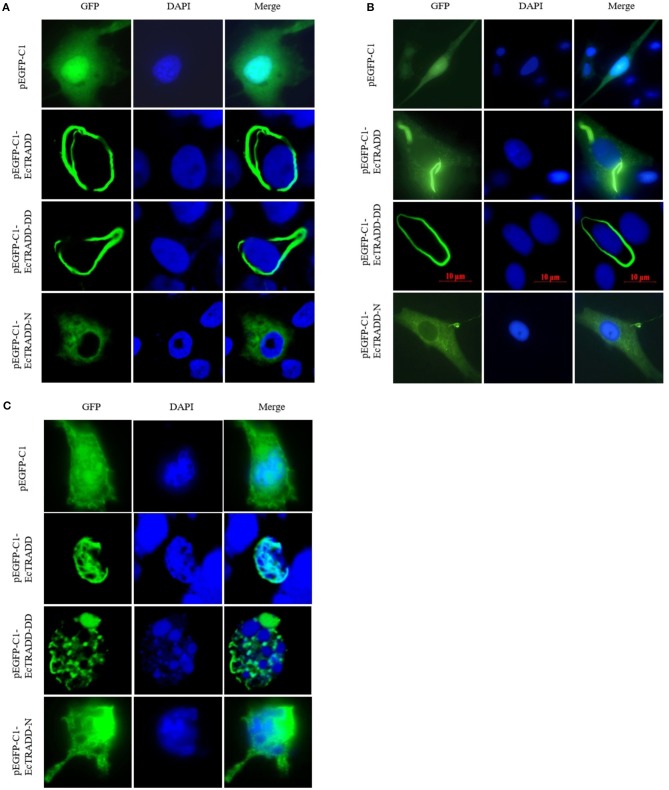
Cellular localization of EcTRADD. **(A)** FHM cells were transfected with pEGFP-C1, pEGFP-EcTRADD, pEGFP-EcTRADD-N, and pEGFP-EcTRADD-DD plasmids using Lipofectamine 2000. Cells were stained with DAPI at 24 h post-transfection and imaged under confocal laser scanning microscopy. **(B)** GS cells were transfected with pEGFP-C1, pEGFP-EcTRADD, pEGFP-EcTRADD-N, and pEGFP-EcTRADD-DD plasmids using Lipofectamine 2000. Cells were stained with DAPI at 24 h post-transfection and imaged under fluorescence microscopy. **(C)** FHM cells were transfected with pEGFP-C1, pEGFP-EcTRADD, pEGFP-EcTRADD-N, and pEGFP-EcTRADD-DD plasmids. Cells were infected with SGIV at 24 h post-transfection, and stained with DAPI at 12 h post-infection, and imaged under confocal laser scanning microscopy.

To investigate the function of *EcTRADD*, the subcellular location was monitored in FHM and GS cells. The fluorescent signals of pEGFP-C1 distributed in both the cytoplasm and the nucleus (Figures 3A,B). Compared with the controls, without SGIV infection, EcTRADD and EcTRADD-DD exhibited a clear pattern of discrete and interconnecting cytoplasmic filaments resembling the death effector filaments (Figures 3A,B), which is similar to the distribution of Bcl10 in FHM cells (Guiet and Vito, 2000; Cottin et al., 2001). In contrast, EcTRADD-N was observed in the cytoplasm (Figures 3A,B). After SGIV infection, the distribution of EcTRADD and EcTRADD-DD transferred to the nucleus (Figure 3C).

### Overexpression of EcTRADD Inhibited SGIV Replication

**Figure 4:**
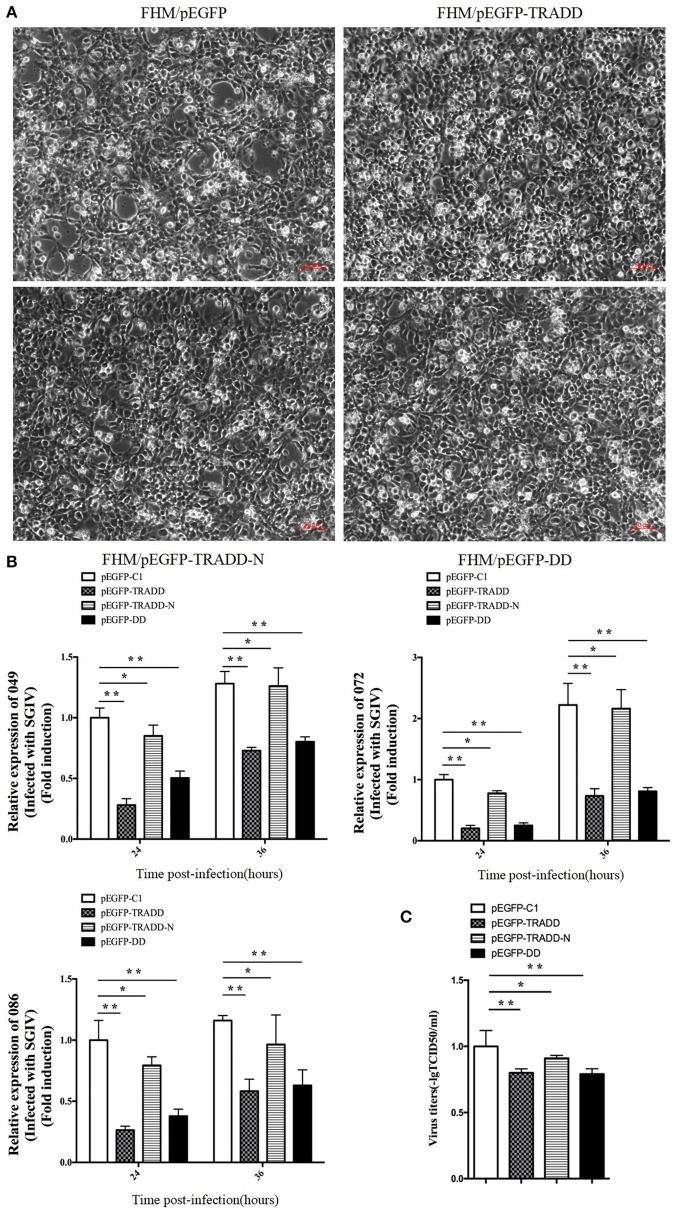
Activities of EcTRADD in SGIV infection and replication *in vitro*. **(A)** Cytopathic effects observed in transfected GS cells infected with SGIV at 24 h p.i. **(B)** Cell were collected for RNA extraction at 24 and 36 h p.i. after SGIV infection. Relative expression levels of SGIV ORF086, ORF049, and ORF072 mRNA were assessed by RT-qPCR and normalized to the reference gene β-actin. **(C)** Virus titers from infected cell lines. Data are represented as mean ± SD (*n* = 3). *P < 0.05; **P < 0.01.

To elucidate the potential roles of EcTRADD on fish virus replication, the transcription level of virus genes in EcTRADD, EcTRADD-N, and EcTRADD-DD overexpressed cells were examined. Severity of the cytopathogenic effect (CPE) evoked by SGIV infection at 24 h p.i. was delayed in cells overexpressing EcTRADD and its domains compared to control cells (Figure 4A). Consistently, the transcription level of SGIV genes (ORF086, ORF049, and ORF072) were significantly decreased relative to the control in EcTRADD, EcTRADD-DD, and EcTRADD-N-overexpressing cells (Figure 4B). Finally, the virus titer assay also indicated that overexpression of EcTRADD and EcTRADD-DD significantly inhibited SGIV replication (Figure 4C). These results indicated that EcTRADD and its domains inhibited the replication of SGIV.

### Effect of EcTRADD on Apoptosis

**Figure 5:**
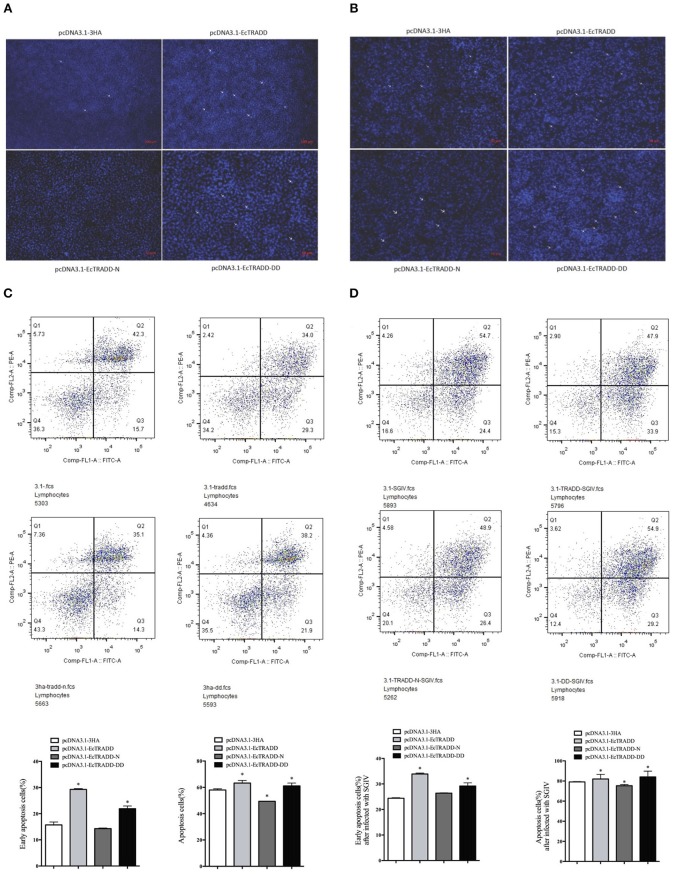
Effect of EcTRADD on apoptosis. **(A)** Cellular nuclear morphology in control cells; arrows indicate the apoptotic bodies. **(B)** Cellular nuclear morphology in SGIV-infected cells; arrows indicate the apoptotic bodies. **(C)** Flow cytometry analysis of DNA content in control cells. **(D)** Flow cytometry analysis of DNA content in SGIV-infected cells. Q3 represents the percentage of early apoptotic cells, and Q2 represents the rate of late apoptotic cells. The differences between early apoptotic cells and total apoptotic cells were analyzed. *P < 0.05.

To assess the effects of EcTRADD and its domains on apoptosis, the eukaryotic expression vectors of pcDNA3.1-EcTRADD, pcDNA3.1-DD, and pcDNA3.1-EcTRADD-N were constructed. Many apoptotic bodies were observed in pcDNA3.1-EcTRADD and pcDNA3.1-EcTRADD-DD-overexpressed cells at 12 h, while few were observed in pcDNA3.1 and pcDNA3.1-EcTRADD-N-overexpressed cells (Figure 5A). SGIV induced typical apoptosis in FHM cells (Huang X. et al., 2011). Then EcTRADD overexpressed cells were infected with SGIV, and the effects of EcTRADD and its domains on apoptosis were measured. The results showed that more apoptotic bodies were observed in SGIV-infected cells (Figure 5B). To further confirm the results, the quantitative analysis by flow cytometry was measured. The data showed that the percentage of apoptotic cells increased in pcDNA3.1-EcTRADD and pcDNA3.1-EcTRADD-DD-overexpressed cells than that in pcDNA3.1-overexpressed cells at 12 h p.i., while it decreased in pcDNA3.1-EcTRADD-N-overexpressed cells (Figure 5C). The percentages of early apoptotic cells were 29.3, 21.9, 14.3, and 15.7% in pcDNA3.1-EcTRADD, pcDNA3.1-EcTRADD-DD, pcDNA3.1-EcTRADD-N, and pcDNA3.1-overexpressed cells, respectively. The total rates of apoptotic cells were 63.3, 60.1, 49.4, and 58% in pcDNA3.1-EcTRADD, pcDNA3.1-DD, pcDNA3.1-EcTRADD-N, and pcDNA3.1-overexpressed cells. When the cells were infected with SGIV, the percentages of early apoptotic cells and the total rate of apoptotic cells all increased (Figure 5D). The percentages of early apoptotic cells increased to 33.9, 29.2, 26.4, and 24.4% in pcDNA3.1-EcTRADD, pcDNA3.1-DD, pcDNA3.1-EcTRADD-N, and pcDNA3.1-overexpressed cells, respectively. While the total rate of apoptotic cells increased to 81.8, 84.1, 75.3, and 79.1% in pcDNA3.1-EcTRADD, pcDNA3.1-DD, pcDNA3.1-EcTRADD-N, and pcDNA3.1-overexpressed cells, respectively.

**Figure 6:**
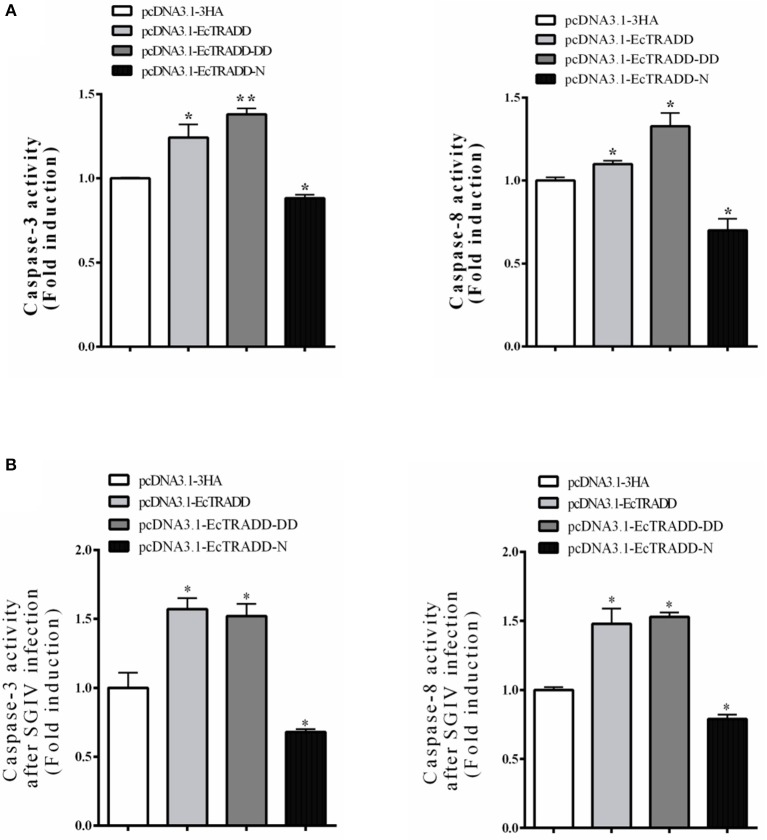
The activities of caspase-3 and caspase-8. **(A)** The activities of caspase-3 and caspase-8 in pcDNA3.1-EcTRADD, pcDNA3.1-EcTRADD-DD, pcDNA3.1-EcTRADD-N, and pcDNA3.1-overexpressed cells without SGIV infection. **(B)** The activities of caspase-3 and caspase-8 in pcDNA3.1-EcTRADD, pcDNA3.1-EcTRADD-DD, pcDNA3.1-EcTRADD-N, and pcDNA3.1-overexpressed cells with SGIV infection. *P < 0.05; **P < 0.01.

Caspase-3 and caspase-8 are key mediators of apoptosis. To evaluate the possible involvement of downstream effectors caspases, the activity of caspase-3 and caspase-8 were detected after EcTRADD and its domains transfection without or with SGIV infection. As shown in Figure 6A, the caspase-3 activity was about 1.24 and 1.38 times higher in pcDNA3.1-EcTRADD and pcDNA3.1-EcTRADD-DD cells than that in pcDNA3.1-overexpressed cells, while it was about 0.8 times lower in pcDNA3.1-N cells than that in pcDNA3.1-overexpressed cells. The caspase-8 activity was about 1.1 and 1.33 times higher in pcDNA3.1-EcTRADD and pcDNA3.1- EcTRADD-DD cells than that in pcDNA3.1-overexpressed cells, while it was about 0.7 times lower in pcDNA3.1-N cells than that in pcDNA3.1-overexpressed cells. When the cells were infected with SGIV, the caspase-3 activity was about 1.48 and 1.53 times higher in pcDNA3.1-EcTRADD and pcDNA3.1-EcTRADD-DD cells than that in pcDNA3.1-overexpressed cells at 6 h after the addition of SGIV, while it was about 0.85 times lower in pcDNA3.1-EcTRADD-N cells than that in pcDNA3.1-overexpressed cells (Figure 6B). The caspase-8 activity was about 1.57 and 1.52 times higher in pcDNA3.1-EcTRADD and pcDNA3.1-EcTRADD-DD cells than that in pcDNA3.1-overexpressed cells at 6 h after SGIV infection at 6 h, while it was about 0.75 times lower in pcDNA3.1-EcTRADD-N cells than that in pcDNA3.1-overexpressed cells (Figure 6B). These results indicate that EcTRADD and EcTRADD-DD induce early cell apoptosis and promote SGIV-induced cell apoptosis, while EcTRADD-N inhibits cell apoptosis.

### Activation of Reporter Genes

**Figure 7:**
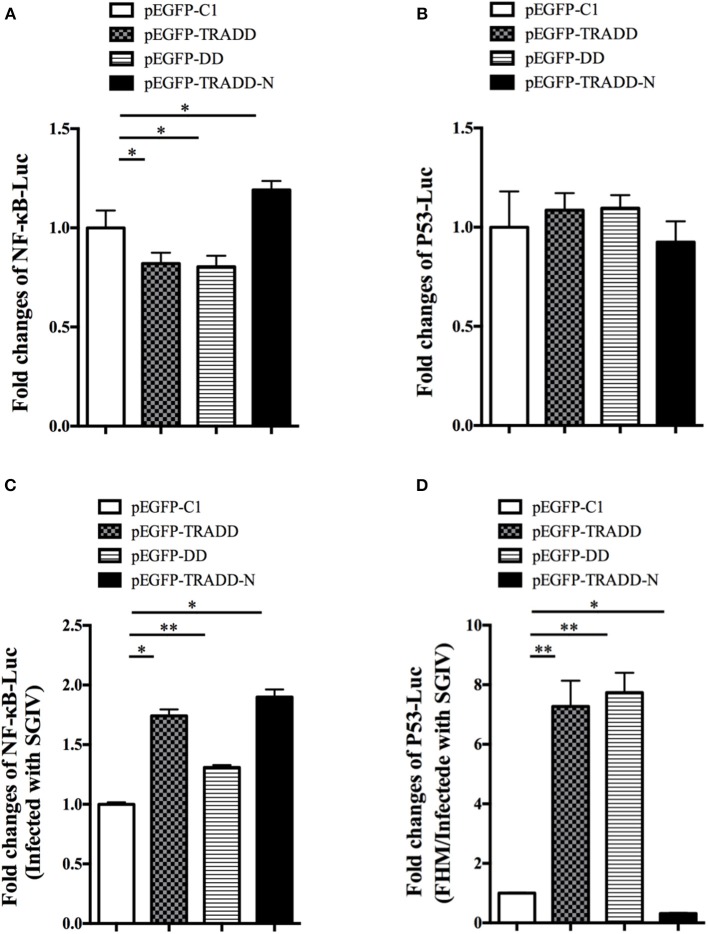
The relative luciferase activities of NF-κB and p53 promoters in EcTRADD-overexpressed cells. **(A)** FHM cells were co-transfected with NF-κB-Luc and pEGFP-EcTRADD or its domains, and then the activities of NF-κB promoters were detected using the luciferase reporter gene assay. **(B)** FHM cells were co-transfected with p53-Luc and pEGFP-EcTRADD or its domains, and then the activities of p53 promoters were detected using the luciferase reporter gene assay. **(C)** FHM cells were co-transfected with NF-κB-Luc and pEGFP-EcTRADD or its domains, and then cells were infected with SGIV at 16 h post-transfection. The activities of NF-κB promoters were detected at 24 h.p.i. using the luciferase reporter gene assay. **(D)** FHM cells were co-transfected with p53-Luc and pEGFP-EcTRADD or its domains, and then cells were infected with SGIV at 16 h post-transfection. The activities of p53 promoters were detected at 24 h.p.i. using the luciferase reporter gene assay. *P < 0.05; **P < 0.01.

To reveal the molecular mechanism of EcTRADD, the activations of reporter genes were investigated in FHM cells. Both EcTRADD and EcTRADD-DD inhibited the activations ofNF-κB in FHM cells, whereas EcTRADD-N promoted its activation. The fold changes were 0.8, 0.8, and 1.2 in cells transfected with pEGFP-EcTRADD, pEGFP-EcTRADD-DD, and pEGFP-EcTRADD-N, respectively (Figure 7A). EcTRADD, EcTRADD-DD, and EcTRADD-N could not affect the activation of p53-Luc (Figure 7B). After SGIV infection, the activations of NF-κB-Luc were increased with fold changes of 1.8, 1.3, and 1.9 in cells transfected with pEGFP-EcTRADD, pEGFP-EcTRADD-DD, and pEGFP-EcTRADD-N, respectively (Figure 7C). P53-Luc was significantly (*P* < 0.01) activated by TRADD and EcTRADD-DD, but suppressed by EcTRADD-N at 24 h p.i., and the fold changes were 7.3, 7.4, and 0.3 in transfected cells, respectively (Figure 7D).

## Discussion

Numerous studies have shown that TRADD is a TNFR1-associated signal transducer and an essential component of the TNFR1 complex that is involved in activating both apoptotic and NF-κB pathways as an adaptor (Olivier and Jürg, 2003). It is also required for TNFR-1-initiated neuronal apoptosis following *in vitro* infection with virus as an essential component of the antiviral response (Zhu et al., 2001; Michallet et al., 2007; Arnd, 2008; Vivek et al., 2010; Bai-Liang et al., 2012; Gaud et al., 2013). However, few studies have focused on the function of TRADD in lower vertebrates and its antiviral response to DNA virus infection. In this study, we cloned and characterized EcTRADD and studied its role and mechanism in the process of fish virus infection.

EcTRADD encodes a 291 amino acid protein that shares 91.81% identity with *P. flavescens* (XP_028437633.1). BLAST analysis indicated that *EcTRADD* covers two conserved domains: the DD and the N-terminal domain. The DD superfamily is a protein-protein interaction domain, including DD, pyrin, CARD (caspase activation and recruitment domain), and DED family (Kohl and Grütter, 2004). DDs as an adapter in the signaling pathway can recruit other proteins into the signaling complex by associating with other members of the DD superfamily (Park et al., 2007). They are important components of the programmed cell death (apoptosis) pathway and are involved in many other signaling pathways like those that affect innate immunity, inflammation, differentiation and cancer (Park et al., 2007). The N-terminal domain folds into an alpha-beta sandwich with a four-stranded beta sheet and six alpha helices, each forming one layer of the structure. The domain allows TRADD to dock onto tumor necrosis factor receptor-associated factor (TRAF), which may be important for direct caspase-8 inhibition and the immediate suppression of apoptosis at the apical point of the cascade, in turn leading to the triggering of NF-κB signaling pathways (Hsu et al., 1996b; Olivier and Jürg, 2003). In this study, nucleus staining, flow cytometry, and caspase activity results showed that overexpression of EcTRADD and EcTRADD-DD promoted apoptosis but inhibited activation of reporter gene NF-κB. Conversely, and overexpression of EcTRADD-N inhibited apoptosis but promoted activation of NF-κB.

Subcellular localization is often important for regulating signaling molecules, so the localization of EcTRADD and its two domains were examined using green fluorescent protein. A recent report suggested that the COOH-terminal portion of TRADD-DD (aa 245–312) may associate with acidic keratins 14 and 18, thereby localizing TRADD to cytoplasmic filaments and decreasing sensitivity to TNF (Inada et al., 2001). Our results showed that EcTRADD-N was excluded from the nucleus and had a diffuse cytoplasmic distribution, whereas both EcTRADD and EcTRADD-DD accumulated in what appeared to be the previously observed DEFs perinuclearly (Siegel et al., 1998; Guiet and Vito, 2000; Michael et al., 2002). Studies have shown that apoptosis triggered by the DED protein motif, which belong to DDs, is in all cases associated with the formation of intracellular DEFs. DEFs can recruit caspase-8 through TRADD, which results in caspase activation and subsequent apoptosis (Siegel et al., 1998). It has been reported that TRADD can shuttle between the cytoplasm and the nucleus (Michael et al., 2002), linking the TNF receptor with p53 and inducing apoptosis by different mechanisms (Guo et al., 2000; Wang et al., 2001; D'Orazi et al., 2002; Michael et al., 2002; Fogal et al., 2014). In this study, the localization of EcTRADD and EcTRADD-DD was transferred to the nucleus after infection with SGIV. Therefore, the localization changes of EcTRADD and EcTRADD-DD after infection with SGIV likely induced apoptosis by different mechanisms than those used in the cytoplasm.

It has been reported that proteins containing the same key interaction domains of DED may have pro- and anti-apoptotic effects. All the apoptosis conditions were related to the formation of death effect filament (DEF). Procaspases are effectively recruited into these structures. Conversely, proteins that inhibit fas-induced apoptosis lack the ability to form these filaments and can antagonize apoptosis by inhibiting the formation of DEF (Jones et al., 2000). After infection with SGIV, DEF seems to be able to act as a bridge to connect TNF receptors in the cytoplasm and nuclear substances such as p53 to transmit information between the cytoplasm and the nuclear. After infection with SGIV, fluorescence localization showed that DEFs could be shuttle-through between nuclei, and the phenomenon of deforming and fragmentation of nuclei could be observed. It was speculated that DEFs might be related to nuclear fragmentation and formation of apoptotic bodies in the process of SGIV-induced apoptosis. Therefore, it is speculated that EcTRADD and its DD domain can induce SGIV-induced apoptosis through the recruitment and activation of procaspase by the formation of cytoplasmic filaments.

The broad antiviral effects of TRADD have been demonstrated in mammalian cells for some viruses (Michallet et al., 2007; Pietras and Genhong, 2008). Based on this information, the expression of EcTRADD was examined under stimulation by SGIV. Expression of EcTRADD was up-regulated after SGIV infection in GS cells. Recently, the viral TRAF protein (ORF111L) from infectious spleen and kidney necrosis virus was found to interact with TRADD and induce caspase 8-mediated apoptosis (Bai-Liang et al., 2012). Therefore, the effect of EcTRADD and its two domains were evaluated on SGIV infection *in vitro*. At 24 h p.i., the severity of CPE induced by SGIV infection was delayed in cells overexpressing EcTRADD, EcTRADD-DD, and EcTRADD-N. Consistently, overexpressing EcTRADD, EcTRADD-DD, and EcTRADD-N reduced the transcription level of three SGIV genes (ORF086, ORF049, and ORF072) at both 24 and 36 h p.i. The infectivity of SGIV was also inhibited by EcTRADD, EcTRADD-DD, and EcTRADD-N. These results suggest that EcTRADD and its two domains all inhibited viral infection and replication.

Viral infections also induce apoptosis (Penny and Tyler, 2009; Kinpara et al., 2013), and apoptosis induced by virus has been found to be related to activation of c-Jun N-terminal kinase, NF-κB, and p53 pathways (Kinpara et al., 2013; Guo et al., 2016a; Zhang et al., 2018). FHM cells infected by SGIV can be induced into typical apoptosis (Huang X. et al., 2011). Research has shown that TRADD can link death receptor devices with nuclear events and induce apoptosis by different mechanisms in the nucleus, which involves p53 and is caspase-independent, and in the cytoplasm, where TRADD induces apoptosis at the TNFR1 (Guo et al., 2000; Wang et al., 2001; D'Orazi et al., 2002; Michael et al., 2002; Fogal et al., 2014). Therefore, the nuclear staining and flow cytometry analysis were conducted, and caspase activities were measured in EcTRADD, EcTRADD-DD, and EcTRADD-N overexpressed cells. The flow cytometry and apoptotic body data showed that both EcTRADD and EcTRADD-DD promoted SGIV-induced cell apoptosis, but EcTRADD-N inhibited it. Both EcTRADD and EcTRADD-DD increased the activities of caspase 3 and caspase 8 in the overexpressed cells without or with SGIV infection, while EcTRADD-N inhibited them. Moreover, EcTRADD and EcTRADD-DD significantly promoted the activation of reporter gene p53 and NF-κB in the overexpressed cells, but EcTRADD-N had no effect to p53.

In summary, EcTRADD was cloned and characterized, and the response of EcTRADD to SGIV challenges was investigated. Intracellular localization was analyzed for EcTRADD and its domains *in vitro*. Overexpression of EcTRADD and its domains inhibited replication of SGIV *in vitro*. Both EcTRADD and EcTRADD-DD induced the caspase-dependent apoptosis, while EcTRADD-N inhibited the apoptosis. Our results firstly demonstrated that the regulatory roles of grouper TRADD in innate immune response during DNA virus infection and will contribute to understanding the function of fish TRADD in response to viruses.

## Data Availability

All datasets generated for this study are included in the manuscript/supplementary files.

## Ethics Statement

The animal study was reviewed and approved by The Animal Care and Use Committee of College of Marine Sciences, South China Agricultural University.

## Author Contributions

QQ and JW designed the experiments. XZ performed the majority of the experiments, analyzed data, and wrote the manuscript. ZL, CL, YZ, and LW contributed experimental suggestions. All authors revised the manuscript.

### Conflict of Interest Statement

The authors declare that the research was conducted in the absence of any commercial or financial relationships that could be construed as a potential conflict of interest.
